# The Association between Red Blood Cell Distribution Width and Mortality Risk after Hip Fracture: A Meta-Analysis

**DOI:** 10.3390/medicina60030485

**Published:** 2024-03-14

**Authors:** Bao Tu Thai Nguyen, Duy Nguyen Anh Tran, Tan Thanh Nguyen, Yi-Jie Kuo, Yu-Pin Chen

**Affiliations:** 1 The International Graduate Program in Medicine, College of Medicine, Taipei Medical University, Taipei 110, Taiwan; d142111005@tmu.edu.tw (B.T.T.N.); d142111006@tmu.edu.tw (D.N.A.T.); 2 Department of Orthopedics, Faculty of Medicine, Can Tho University of Medicine and Pharmacy, Can Tho 900000, Vietnam; nttan@ctump.edu.vn; 3 Department of Orthopedics, Wan Fang Hospital, Taipei Medical University, Taipei 116, Taiwan; benkuo5@tmu.edu.tw; 4 Department of Orthopedics, School of Medicine, College of Medicine, Taipei Medical University, Taipei 110, Taiwan

**Keywords:** hip fracture, red blood cell distribution width, mortality, prognosis, meta-analysis, chronic inflammation, oxidative stress, bone healing, fragility

## Abstract

*Background and Objectives*: Hip fractures in the elderly pose a considerable health risk and cause concern. Red blood cell distribution width (RDW) is a valuable marker for identifying patients at high risk of age-related mortality and various disorders and diseases. However, its association with poor patient outcomes following hip fractures has yet to be fully established. Hence, the purpose of this meta-analysis was to investigate and gain a better understanding of the relationship between RDW levels and the risk of mortality after hip fractures. *Materials and Methods*: PubMed, Embase, Web of Science, and other databases were comprehensively searched until April 2023 to identify relevant studies. The meta-analysis included observational studies finding the association between RDW at admission or preoperation and short-term and long-term mortality rates following hip fractures. The results were presented in terms of odds ratios (ORs) or hazard ratios (HRs) with corresponding 95% confidence intervals (CIs). *Results*: This meta-analysis included 10 studies involving 5834 patients with hip fractures. Patients with preoperative RDW of over 14.5% had higher risks of 1-year (OR: 5.40, 95% CI: 1.89–15.48, *p* = 0.002) and 3-month (OR: 2.91, 95% CI: 1.42–5.95, *p* = 0.004) mortality. Higher admission or preoperative RDW was significantly associated with an 11% higher mortality risk after 1 year (HR: 1.11, 95% CI: 1.06–1.17, *p* < 0.00001). Patients with higher preoperative RDW had a significantly higher risk of 6-month mortality, which was three times that of those with lower preoperative RDW (OR: 3.00, 95% CI: 1.60–5.61, *p* = 0.0006). Higher preoperative RDW was correlated to a higher 30-day mortality risk (OR: 6.44, 95% CI: 3.32–12.47, *p* < 0.00001). *Conclusions*: Greater RDW values at admission or before surgery were associated with a higher risk of short-term and long-term mortality following hip fractures. Because RDW can be easily measured using a routine blood test at a low cost, this parameter is promising as an indicator of mortality in elderly patients with hip fractures.

## 1. Introduction

Hip fractures commonly affect individuals over the age of 65 years and can pose physical impairment and care reliance, reduced quality of life, increased risks of complications, and even death [1]. By 2025, there will be 2.6 million hip fracture patients, and by 2050, that number will tremendously hit 6.26 million [2]. Moreover, hip fractures and their associated health problems impose a considerable burden on caregivers and communities, with the corresponding annual cost to the US healthcare system being 5.96 billion US dollars [3]. Despite advances in surgical methods and multidisciplinary care, hip fractures are also associated with a substantial mortality risk, which has been reported to range from 10% at a 1-month follow-up time point to 36% at a 1-year follow-up time point [2,4,5,6,7]. Considering this high mortality rate, high-risk patients must be identified to facilitate effective decision making regarding treatment courses, rehabilitation programs, long-term care strategies, and preventative measures [8].

Studies have revealed a potential link between blood markers obtained via routine blood tests and hip fracture outcomes. Specifically, studies have reported that low hemoglobin, low albumin, high creatinine, and high C-reactive protein (CRP) were associated with a relatively high risk of death [9,10]. Moreover, red blood cell distribution width (RDW) has been identified as a probable predictor for mortality following hip fractures [11,12,13,14]. RDW is a measure of the heterogeneity in the size of red blood cells (RBCs) and is derived from a complete blood count test. Expressed as a percentage, RDW is determined by dividing the standard deviation of RBC volume by the mean corpuscular volume. Obtaining RDW from a complete blood count test is straightforward [15,16]. The average volume of RBCs in humans is 80 to 100 fL, and the normal RDW range is 12% to 15%; the range varies by test laboratory and population [17,18].

While RDW values below the normal range are rare and less significant in clinical practice, high RDW has raised more concerns. High RDW has traditionally been primarily seen in diagnosing anemia caused by a lack of iron, folate, or vitamin B12. The latest breakthrough revealed that RDW can also serve as a valuable tool for identifying patients at high risks of aging-related mortality and various disorders or diseases—including cardiovascular disease, colon cancer, diabetes mellitus, kidney or liver diseases, and pulmonary disease—and for identifying patients requiring intensive care [15,19,20]. Several studies in the field of orthopedics and trauma have also linked RDW, alone or in conjunction with other factors, to frailty, complications after orthopedic surgery, and specific types of fractures [21,22,23,24]. However, no definitive evidence on the predictive significance of RDW for hip fracture mortality has been shown.

To address the existing literature’s limited and inconclusive findings, we conducted a meta-analysis to investigate the relationship between RDW value at admission or preoperation and the long- and short-term mortality rates in patients who have experienced hip fractures. Our hypothesis posits that an elevated RDW level is associated with an increase in mortality risk after a hip fracture.

## 2. Materials and Methods

This study reported using the Preferred Reporting Items for Systematic Reviews and Meta-Analyses guideline [25] and referred to the guidance from the *Cochrane Handbook for Systematic Reviews of Interventions* [26]. It was registered with the International Prospective Register of Systematic Reviews and was assigned the identification number CRD42023427458 [27].

### 2.1. Search Strategy and Study Identification 

Two independent reviewers (B.T.T. Nguyen and D.N.A. Tran) comprehensively searched the PubMed, Embase, and Web of Science electronic databases for titles, abstracts, and full-text articles. The search involved the use of keywords such as “RDW”, “red cell distribution width”, “red blood cell distribution width”, and “routine blood test” combined with terms related to “hip fracture”, “risk”, “prognosis”, “predictor”, “mortality”, and “survival”. The search covered all publications available until April 2023. To gather additional relevant articles, the reviewers further performed a search of Google Scholar, ResearchGate, and the reference lists of the included articles. Any discrepancies were addressed to the third author (Y.P. Chen) for conclusion. The reviewers used EndNote version 20.5 (Clavirate, London, UK) to manage the retrieved studies, remove duplicate ones, and manage the references.

### 2.2. Inclusion and Exclusion Criteria

Prospective or retrospective cohort or case–control studies were included in this study. The target population comprised patients with hip fractures of any type (femoral head, femoral neck, or trochanteric fractures). RDW values were collected from patients’ routine blood tests at admission or before surgery. Based on the RDW cut-off value used in each study, the patients were classified into high RDW and normal RDW groups. The investigating outcomes were 30-day, 3-month, 6-month, and ≥1-year mortality. Furthermore, we excluded cross-sectional studies, case reports, case series, expert opinions, reviews, letters, systematic reviews, and studies for which the full text or data necessary to our study were unavailable. The two reviewers independently assessed titles and abstracts to determine suitable studies. We did not impose restrictions on language or publication year. The full texts were collected and evaluated for inclusion. The third author resolved any disagreements.

### 2.3. Data Extraction and Methodological Quality

The two reviewers independently reviewed relevant studies and extracted data from the provided materials. The retrieved data included each study’s publication year, nation, research design, and follow-up time points; models used to predict mortality following hip fractures; and the number of participants, mean age, sex distribution, and RDW values of survivors and nonsurvivors after hip fractures. Any discrepancies were addressed to the third author for resolution.

The Newcastle–Ottawa Scale (NOS) was used to rate the quality of the included non-randomized studies [28]. The NOS had two quality assessment scales for case–control studies and cohort studies. The two reviewers evaluated the quality of each study by considering three assessment categories: selection domain, comparability domain, and outcome/exposure domain. In the event of disagreements, the reviewers consulted a third reviewer to make a final decision.

### 2.4. Outcomes of Interest

The primary outcome of the study was the association of preoperative or admission RDW values with long-term all-cause mortality (assessed after a ≥1-year follow-up period) and short-term all-cause mortality (set at 30-day, 3-month, and 6-month follow-up time points) among patients who had experienced hip fractures.

### 2.5. Statistical Analysis, Data Synthesis, and Heterogeneity Assessment

We performed our meta-analysis using Review Manager software (RevMan) [Computer program] (version 5.4; The Cochrane Collaboration 2020, Oxford, UK). To synchronize the data for analysis, we transformed, when possible, the data obtained from the included studies by converting median and range values into mean and standard deviation values. This was carried out using formulas derived from previous research [29]. Continuous variables were presented as mean and standard deviation. The outcome measures from each study were combined in the analysis. Odds ratio (OR) and 95% confidence interval (CI) were used as summary statistics for dichotomous outcomes and given odds ratios. Hazard ratio (HR) and 95% CI were also used as summary statistics for studies that provided HRs. Heterogeneity across the included studies was evaluated using the Chi-square test, with the significance set at a *p*-value below 0.1, and the Higgins I^2^ test, with I^2^ values under 25%, indicating low heterogeneity, in the range of 25–50%, indicating moderate heterogeneity, and exceeding 50% indicating significant heterogeneity [30]. If no significant heterogeneity was detected, a fixed-effects model was used. In the case of significant heterogeneity (*p* < 0.1 and I^2^ > 50%), a random-effects model was chosen. The meta-analysis findings were visualized using a forest plot. Additionally, we conducted a sensitivity analysis by excluding one study by one in case of significant heterogeneity between studies. A *p*-value below 0.05 determined statistical significance. 

## 3. Results

**Figure 1:**
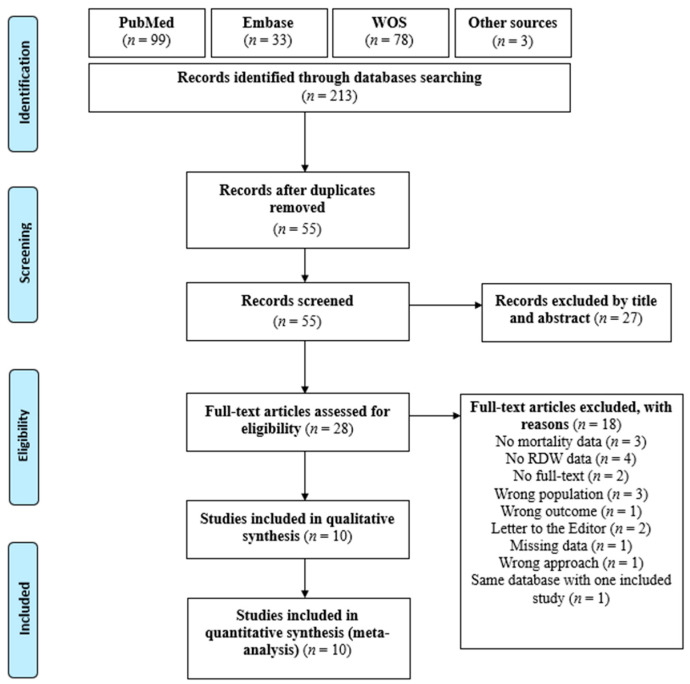
The study screening and selection process flow chart.

Figure 1 illustrates a flow chart of the study screening and selection process. A total of 210 studies were collected from the PubMed, Embase, and Web of Science databases without any language restrictions. Three additional studies were obtained from Google Scholar and ResearchGate. After the removal of duplicates, 55 studies remained; the titles and abstracts of these studies were screened, and 27 of the studies were subsequently removed. The remaining 28 studies were subjected to a full-text screening process to determine their eligibility. After assessing for eligibility, 18 studies were excluded because they did not have full text (*n* = 2), did not have mortality data (*n* = 3), did not have RDW data (*n* = 4), did not include our target population (*n* = 3), did not assess our outcome of interest (*n* = 1), involved letters to editors (*n* = 2), had missing data (*n* = 1), used an irrelevant study design or approach (*n* = 1), or shared a database with another study that was included in the review (*n* = 1). As a result, 10 studies remained to perform the meta-analysis [11,12,13,14,31,32,33,34,35,36].

Supplementary Table S1 provides an overview of the included studies. The 10 studies were conducted between 2013 and 2022 in Türkiye [31,33,34,36], Jordan [32], China [12,35], the United Kingdom [11], Israel [13], and Peru [14], involving a total of 5834 patients (2241 men and 3593 women) with a pooled mean age of 87.61 ± 3.21 years. These studies comprised four retrospective cohort studies [13,32,33,36], two retrospective case–control studies [31,34], three prospective cohort studies [11,12,35], and one retrospective and prospective cohort study [14]. Eight studies indicated that all patients included in their analyses underwent surgery after sustaining hip fractures [11,13,14,32,33,34,35,36], one study reported that 113 of 1479 patients received conservative treatment only [12], and one study did not provide any information regarding post-fracture interventions [31].

The methodological quality of the included studies was evaluated via the modified NOS. Among eight cohort studies, three studies [14,33,36] did not report the confounders in their multivariable analyses, and two studies [33,36] did not describe the ascertainment of exposure and how to assess the outcome. Two case–control studies [31,34] clearly demonstrated their process of selecting participants, identifying exposures, and providing the factor utilized for matching. Supplementary Tables S2 and S3 present the assessment results in detail.

### 3.1. Association between RDW and 1-Year Mortality

**Figure 2:**
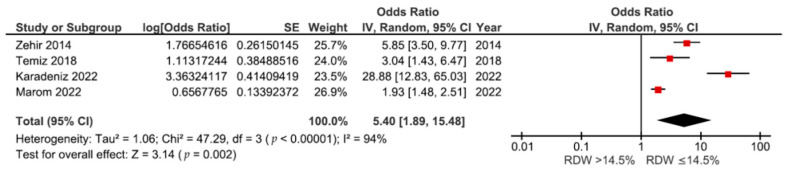
Forest plot of the association between RDW and 1-year mortality risk after hip fractures [13,33,34,36].

Of the ten studies, four [13,33,34,36], including 2246 patients with hip fractures, demonstrated the association between preoperative RDW and 1-year mortality risk amongst the patients. As shown in Figure 2, RDW values of over 14.5% were correlated with a higher risk of mortality than RDW values of ≤14.5% (OR: 5.40, 95% CI: 1.89–15.48, *p* = 0.002). Significant heterogeneity was observed across the analyzed studies (I^2^: 94%, *p* < 0.00001).

Furthermore, Supplementary Figure S1 presents the meta-analysis results for the hazard ratio of over-1-year mortality, combining data from three studies [11,12,31] that involved 2737 patients with hip fractures. The analysis revealed a significant association, indicating that patients with higher RDW had an 11% increased mortality risk (HR: 1.11, 95% CI: 1.06–1.17, *p* < 0.00001) compared with lower RDW values. Low heterogeneity was observed among the analyzed studies (I^2^: 25%, *p* = 0.26).

### 3.2. Association between RDW and 6-Month Mortality

**Figure 3:**
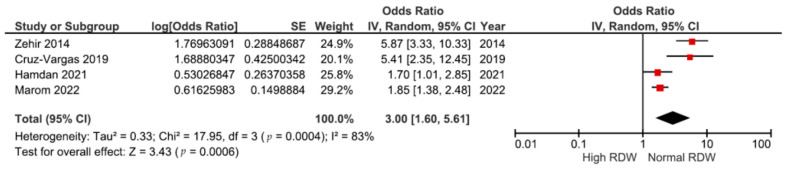
Forest plot of the association between RDW and 6-month mortality risk after hip fractures [13,14,32,36].

Four studies [13,14,32,36], including 2538 patients with hip fractures, showed the association between preoperative RDW and 6-month mortality risk. The pooled odds ratio in Figure 3 reveals a significant increase of three times in the mortality risk in the higher RDW level group (OR: 3.00, 95% CI: 1.60–5.61, *p* = 0.0006). The studies exhibited significantly high heterogeneity (I^2^: 83%, *p* = 0.0004). 

### 3.3. Association between RDW and 3-Month Mortality

**Figure 4:**
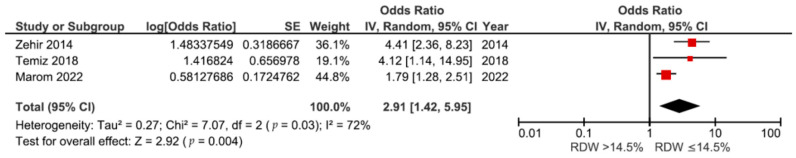
Forest plot of the association between RDW and 3-month mortality risk after hip fractures [13,34,36].

Three studies [13,34,36], including 2056 patients with hip fractures, investigated the effects of RDW levels on 3-month mortality following hip fractures. As presented in Figure 4, RDW values of over 14.5% were associated with a significantly higher risk of 3-month mortality than RDW values of ≤14.5% (OR: 2.91, 95% CI: 1.42–5.95, *p* = 0.004). The studies exhibited significant heterogeneity (I^2^: 72%, *p* = 0.03).

### 3.4. Association between RDW and 30-Day Mortality

**Figure 5:**
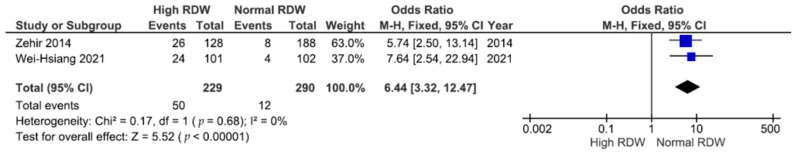
Forest plot of the association between RDW and 30-day mortality risk after hip fractures [35,36].

Two studies [35,36], including 519 patients with hip fractures, determined the association between preoperative RDW and 30-day mortality risk. As displayed in Figure 5, higher preoperative RDW values were related to a higher risk of 30-day mortality when compared with lower RDW values (OR: 6.44, 95% CI: 3.32–12.47, *p* < 0.00001). No heterogeneity was observed between the studies (I^2^: 0%, *p* = 0.68).

### 3.5. Sensitivity Analysis

We conducted a sensitivity analysis to assess studies influencing the pooled results. As presented in Supplementary Figure S2, after the removal of Karadeniz’s study [33], the OR for the association between RDW and 1-year mortality risk became 3.19 (95% CI: 1.50–6.80), and the heterogeneity among the remaining studies was significantly high (I^2^: 86%, *p* = 0.0007). As displayed in Supplementary Figure S3, after the removal of Zehir’s study [36] from analysis, the pooled OR for the association between RDW and 6-month mortality risk became 2.28 (95% CI: 1.36–3.84, *p* = 0.002), and the heterogeneity among the remaining studies was acceptable (I^2^: 67%, *p* = 0.05). 

## 4. Discussion

This meta-analysis included ten studies with a total of 5834 hip fracture patients. We identified significant associations between admission or preoperative RDW with 30-day, 3-month, 6-month, and ≥1-year mortality risks following hip fractures. Our findings demonstrate that hip fracture patients presenting greater RDW values at admission or before surgery are associated with considerably higher risks of mortality after fractures.

RDW is a measure of the variation in the size of RBCs, and it can be easily derived from a complete blood count test. Although it is uncommon to encounter RDW values below the normal range in clinical settings, and their significance is questionable, values exceeding the normal range, which suggest significant variability in RBC size, are frequently seen in various physiological and pathological conditions [15]. The literature has revealed that oxidative stress and inflammation considerably increase RBC heterogeneity, thus increasing RDW values [37,38]. High RDW values were reported to be significantly associated with elevated high-sensitivity CRP levels and erythrocyte sedimentation rates—two frequently used markers of inflammation—in unselected inpatients; moreover, high RDW values might indicate selenium deficiency, which can lead to reduced glutathione peroxidase activity in RBCs and can thus engender erythrocyte deformity [39]. Accordingly, chronic inflammation and oxidative stress are detrimental to homeostasis and shorten RBC survival. Other potential causes of high RDW values are nutrition deficiency, erythrocyte fragmentation, and erythropoietin production dysfunction [15]. Furthermore, studies have reported high RDW values related to acute diabetic ketoacidosis and vascular complications in patients with type 2 diabetes or an increased cardiovascular risk related to atherosclerosis [18,40,41,42]. High RDW values were reported to be correlated to morbidity, all-cause, and specific-cause mortality risks. Beyond merely serving as a prognostic marker for mortality in conditions such as heart disease, kidney disease, and cancer, the significance of RDW extends further. It encompasses the assessment of inflammation levels in conditions like sepsis and autoimmune diseases and the evaluation of infection severity, and it may also offer insights into the presence of neurological or liver disorders [15,17,19,20,43,44,45,46]. 

Hip fractures are severe injuries, especially when they occur in elderly patients. Hip fracture most frequently occurs in patients over 65 [1,47], and the literature indicates that RDW increases with age [15]. Patel et al. demonstrated that RDW is a valuable parameter for effectively predicting all-cause or cause-specific mortality in adults aged ≥45 years [20]. All our included studies showed hip fracture patients with an extremely high pooled mean age of 87.61 years, with the highest mean age [13] being 90.77 and the lowest mean age [35] being 71.75. Screening for frailty in older patients is necessary because patients with frailty are more vulnerable to adverse outcomes when hospitalized. These patients frequently have a combination of acute conditions and comorbidities, which can worsen their health. Because of this increased vulnerability, this population has a relatively high mortality rate [48]. 

The human body reacts to fractures by triggering immune inflammation and oxidative stress [49]. The inflammatory phase after a fracture is crucial to the healing process. This phase must be appropriately controlled to ensure adequate bone healing. However, older age can disturb this regulation, prolonging the inflammatory phase and adversely affecting bone healing and patient recovery [50]. Oxidative stress can occur via an ischemia-reperfusion mechanism in the stage of the fracture healing process when there are increasing numbers of vessels and inflammatory cells formed in the callus [51]. Patients who undergo hip fracture surgery or are discharged exhibit relatively high RDW values, strongly associated with a relatively high mortality risk. Relevant studies have provided substantial evidence supporting the link between higher RDW values in these patients and higher mortality risks [52,53]. This suggests that hip fracture patients hospitalized with initially high RDW values may experience a subsequent increase in RDW because of factors such as inflammation and oxidative stress. Other hypotheses have been described in previous papers, including bone marrow disorder, clonal hematopoiesis, poor renal function, and abnormalities of erythropoietin response, which could elevate the RDW value [34,38]. The increase in RDW could be a key factor to indicate a greater risk of complications and mortality in these patients. The complex interplay between inflammation, oxidative stress, and physiological responses to hip fractures may underlie this association. Further investigation of these mechanisms is warranted to understand better the relationship between high RDW values and unfavorable outcomes in this population.

In clinical practice, surgeons frequently use hemoglobin for managing and forecasting the prognosis of individuals with hip fractures. However, prior studies have indicated that while hemoglobin is valuable for predicting the likelihood of transfusions or poor functional outcomes, its ability to forecast mortality risk is limited to acute situations or in-hospital settings rather than over the long term [54,55,56]. Our study suggests that RDW can be a useful parameter for not only short-term but also long-term mortality in patients with hip fractures.

Until now, hip fracture management guidelines have not typically recognized RDW as a formal indicator of poor prognosis. Instead, these guidelines have focused on clinical factors like age, pre-existing health conditions, functional status before the fracture, the type of fracture, the timing of surgical intervention, and hemoglobin as critical determinants of outcomes in hip fracture patients [57,58]. Therefore, it is advisable to incorporate RDW as a significant predictor in the guidelines for managing hip fractures.

There should be a discussion of some of the limitations of this study. First, data on specific-cause mortality were insufficient; thus, we focused on all-cause mortality instead. Although data on all-cause mortality are useful, we need more data on specific-cause mortality to investigate the relationship between RDW and specific-cause mortality. Hence, future research should focus on specific-cause mortality to help better understand the association between RDW and mortality risk in patients with hip fractures. Second, the limited number of incorporated studies and their associated sample sizes may compromise the study’s robustness. This limitation could hinder the formal assessment of the analyses and also impede the ability to evaluate publication bias [26]. Third, the included studies differed in terms of how they reported RDW values. Specifically, the RDW cut-off values varied among the included studies. Although the studies applied a specific RDW cut-off of 14.5% for their 1-year and 6-month mortality analyses, they did not use standardized cut-off values in their other analyses. RDW cut-offs can generally vary depending on recommendations from individual laboratories or centers. Therefore, a more appropriate approach may entail categorizing RDW as high or normal rather than using specific cut-off values. Such a flexible approach allows for the consideration of context-specific recommendations and facilitates the interpretation and applicability of the findings across different settings. Fourth, because the types of data provided varied across the included studies, we had to standardize the data via some mathematical modifications in the data synthesis process before using them in our analyses, which may have introduced bias to the results. Considering the potential impact of data transformation on the overall conclusions drawn in the study, we advise caution in interpreting our findings. Fifth, the risk of bias assessment of the included studies revealed three studies because they did not clearly report the confounders in their multivariate analyses. This underscores the significance of accurately defining and addressing confounders in such analyses, which could enhance our understanding of the impact of RDW on prognosis. Finally, the results demonstrated high heterogeneity since the number of studies analyzed in each outcome was small, and there was variation in the reported outcome and study designs. This heterogeneity might also be affected by different mortality rates within each study or the co-existence of morbidities in the patient cohort. 

Studies have shown a consistent association between elevated RDW levels and increased mortality after hip fracture. This association is potentially due to RDW reflecting underlying chronic inflammation, nutritional deficiencies, or impaired bone marrow function, all of which contribute to poorer outcomes [38]. With the above results, the initial clinical implications are promising. However, further research is necessary to understand the RDW value fully. Mechanistic studies can shed light on the exact biological pathways linking RDW to mortality, paving the way for targeted therapies aimed at addressing these underlying conditions. Additionally, prospective studies are crucial to confirm the long-term predictive value of RDW, define a universal cut-off, and assess its effectiveness across diverse populations. Furthermore, combining RDW with established risk factors or novel biomarkers might create a more robust risk stratification tool, allowing for even more precise identification of high-risk patients. Moreover, cost-effectiveness analysis is essential to ensure that incorporating RDW into routine clinical practice offers tangible benefits to healthcare systems. By elucidating the biological mechanisms, optimizing its integration into clinical workflows, and demonstrating its cost-effectiveness, RDW has the potential to become a powerful tool for guiding clinical decision making and improving outcomes for not only hip fracture patients but also fragility fracture patients. Implementing this marker holistically, alongside other clinical assessments and patient characteristics, can pave the way for personalized treatment strategies and ultimately improve the patients of those facing this challenging medical condition.

This study demonstrates the most comprehensive meta-analysis to explore the association between RDW levels and mortality rates following hip fracture. The findings indicate that patients with greater RDW values at admission or prior to surgery tend to have a higher risk of both short-term and long-term mortality. Accordingly, RDW can serve as a valuable indicator of mortality in individuals who have experienced a hip fracture and enable potentially guiding treatment decisions to improve patient outcomes. 

## 5. Conclusions

This meta-analysis demonstrated that high RDW values upon admission or before surgery were associated with higher short-term and long-term mortality risks following hip fractures. Because RDW can be easily measured using a routine blood test at a low cost, this parameter is promising as an indicator of mortality in elderly patients with hip fractures.

## Data Availability

All data analyzed in this study are included in these published articles [11,12,13,14,31,32,33,34,35,36].

## Supplementary Materials

The following supporting information can be downloaded at https://www.mdpi.com/article/10.3390/medicina60030485/s1; Table S1: Characteristics of included studies, Table S2: Risk of bias assessment for cohort studies (Newcastle-Ottawa Scale), Table S3: Risk of bias assessment for case-control studies (Newcastle-Ottawa Scale), Figure S1: Forest plot of the association between RDW and >1-year mortality risk after hip fractures, Figure S2: Forest plot of the association RDW and 1-year mortality risk following hip fractures after sensitivity analysis, Figure S3: Forest plot of the association between RDW and 6-month mortality risk following hip fractures after sensitivity analysis.
