# Supplementary material for: The Association between Red Blood Cell Distribution Width and Mortality Risk after Hip Fracture: A Meta-Analysis
Source: Medicina (Kaunas). 2024 Mar 14;60(3):485. doi: 10.3390/medicina60030485 (PMC10972257; doi:10.3390/medicina60030485)
Supplement: Supplementary file 1 [file medicina-60-00485-s001.zip › medicina-2871162-supplementary.pdf]

The Association Between Red Blood Cell Distribution Width and Mortality Risk After Hip Fractures: A Meta-Analysis

SUPPLEMENTARY MATERIALS

Supplementary Tables

Table S1. Characteristics of included studies

No.	Author (year)	Country	Design	Model	Sample size n = 5834	Women n = 3593	Cut-off	Mean age (years) 87.61 ± 3.21	Follow-up timepoint	Confounders/Matching	HR provided
1	Cruz-Vargas (2019)[14]	Peru	Retrospective and prospective cohort	Preoperative RDW	99	64	14.1%	83.15<br>D: NA<br>S: NA	6 months	NR	No
2	Emektar (2017)[31]	Türkiye	Retrospective case–control	Admission RDW	560	348	NR	78.99<br>D: 81.68<br>S: 78.69	1 year	Age, sex, comorbidities, hemoglobin level, WBC count, lymphocyte count, neutrophil count, MPV, RDW, and type of fracture	Yes
3	Garbharran (2013)[11]	United Kingdom	Prospective cohort	Preoperative RDW	698	465	Fourth quartile	78<br>D: NA<br>S: NA	1 year	Admission RDW, Hb, MCV, age, sex, prefracture residence, supporting indoor mobilization, ASA ≥3, CCI, postoperative delirium, cardiac, respiratory and GI complications, serum creatinine >120mmol/l	Yes
4	Hamdan (2021)[32]	Jordan	Retrospective cohort	Preoperative RDW	549	274	15%	76.42<br>D: 79.76<br>S: 75.94	6 months	Age, sex, RDW grouped values, type of fracture, cardiovascular disease	No
5	Karadeniz (2022)[33]	Türkiye	Retrospective cohort	Preoperative RDW	190	127	14.5%	82.8<br>D: 84.2<br>S: 82.1	1 year	NR	No

6	Lv (2015)[12]	China	Prospective cohort	Admission RDW	1479	864	Fourth quartile	73.75<br>D: NA<br>S: NA	2 years	Age, prior myocardial infarction, chronic renal failure, ASA score, treatment, in-hospital pneumonia, and in-hospital circulatory complication	Yes
7	Marom (2022)[13]	Israel	Retrospective cohort	Preoperative RDW	1574	1082	14.5%	90.77<br>D: NA<br>S: NA	1 year<br>6 months	Comorbidities, serum analysis findings, surgeon's seniority	No
8	Temiz (2018)[34]	Türkiye	Retrospective case–control	Preoperative RDW	166	97	14.5%	79.16<br>D: 84.96<br>S: 79.5	1 year<br>3 months	Mortality status	No
9	Wei-Hsiang (2021)[35]	China	Prospective cohort	Preoperative RDW	203	89	13.35%	71.75<br>D: 72.04<br>S: 71.7	30 days	Multivariate Cox proportional Hazard analysis using age and gender for model A or age, gender, BMI, SBP, DBP, diabetes, hypertension, smoking, and drinking for model B	Yes
10	Zehir (2014)[36]	Türkiye	Retrospective cohort	Preoperative RDW	316	183	14.5%	77.86<br>D: 84.96<br>S: NA	1 year<br>6 months<br>3 months<br>30 days	NR	No

RDW: red blood cell distribution width; NR: not reported; HR: hazard ratio; D: death; S: survival, WBC: White blood cell, MPV: Mean platelet volume, ASA: American Society of Anaesthesiologists, CCI: Charlson comorbidity index, GI: Gastrointestinal, BMI: Body mass index, SBP: systolic blood pressure, DBP: diastolic blood pressure,

Table S2. Risk of bias assessment for cohort studies (Newcastle-Ottawa Scale)

Study	Selection				Comparability	Outcome		
	Representativeness of Exposed Cohort	Selection of the Non-exposed cohort from the same source as the exposed cohort	Ascertainment of Exposure	Outcome of interest was not present at Start of Study	Comparability of cohorts	Assessment of outcome	Follow-up long enough for outcome to occur	Adequacy of follow-up
Cruz-Vargas (2019)[14]	Participants were truly representative of elderly patients with hip fractures among the community.	Yes	Hospital records	Yes	Cox multivariate regression was performed. Confounders were not clearly reported	Independent blind assessment	Yes	100% complete follow-up
Garbharran (2013)[11]	Participants were truly representative of patients with hip fractures among the community.	Yes	Secure records	Yes	Admission RDW, Hemoglobin, MCV, age, sex, prefracture residence, supporting indoor mobilization, ASA score ≥3, CCI, postoperative delirium, cardiac, respiratory, and GI complications, serum creatinine >120mmol/l for Cox proportional hazards multivariate analysis	Independent blind assessment	Yes	100% participants in 1-year follow-up
Hamdan (2021)[32]	Participants were truly representative of patients with hip fractures among community.	Yes	Medical records	Yes	Age, gender, RDW grouped values, type of fracture, and the presence of cardiovascular disease for multivariate regression analysis	Independent blind assessment	Yes	92.3% of participants were included after excluding missing medical records, follow-up loss, and younger than 50 years
Karadeniz (2022)[33]	Participants were truly representative	Yes	No description	Yes	Multivariate logistic regression was performed.	No description	Yes	Complete follow-up

	of patients with hip fractures among community.				Confounders were not clearly reported.			
Lv (2015)[12]	Participants were truly representative of patients with hip fractures among the community.	Yes	Hospital database	Yes	Age, prior myocardial infarction, chronic renal failure, ASA score, treatment, in-hospital pneumonia, and in-hospital circulatory complication for Multivariable Cox hazardous proportional analysis	Telephone or medical records	Yes	87.77% of participants were followed up at the final time point
Marom (2022)[13]	Participants were truly representative of patients with hip fractures among community.	Yes	Medical records	Yes	Comorbidities, serum analysis, surgeon seniority for multivariate logistic regression model	Independent blind assessment	Yes	91.24% of participants were included after excluding 151 missing data
Wei-Hsiang (2021)[35]	Participants were truly representative of patients with hip fractures among the community.	Yes	Medical records	Yes	Multivariate Cox proportional Hazard analysis using age and gender for model A or age, gender, BMI, SBP, DBP, diabetes, hypertension, smoking, and drinking for model B	Independent blind assessment	Yes	60.78% of participants complete the follow-up with a detailed description of follow-up loss
Zehir (2014)[36]	Participants were truly representative of patients with hip fractures among community.	Yes	No description	Yes	Not reported	No description	Yes	Complete follow-up

RDW: red blood cell distribution width, MCV: mean corpuscular volume, ASA: American Society of Anaesthesiologists, CCI: Charlson comorbidity index, GI: Gastrointestinal, BMI: Body mass index, SBP: systolic blood pressure, DBP: diastolic blood pressure

Table S3. Risk of bias assessment for case-control studies (Newcastle-Ottawa Scale)

**Study**	**Selection**				**Comparability**	**Exposure**		
	**Is the case definition adequate?**	**Representativeness of the cases**	**Selection of controls**	**Definition of controls**	**Comparability of cases and controls**	**Ascertainment of exposure**	**Same method of ascertainment for cases and controls**	**Non-response rate**
Emektar (2017)[31]	Yes	Consecutive representative series of cases	Hospital controls	Yes	Mortality vs survival at one-year follow-up<br><br>Age, sex, comorbidities, hemoglobin, WBC count, lymphocyte count, neutrophil count, MPV, RDW, type of fracture for multivariate Cox regression model	Hospital database	Yes	Non respondents described
Temiz (2018)[34]	Yes	Consecutive representative series of cases	Hospital controls	Yes	Mortality status	Medical records	Yes	Non respondents described

WBC: White blood cell, MPV: mean platelet volume, RDW: Red blood cell distribution width

Supplementary Figures

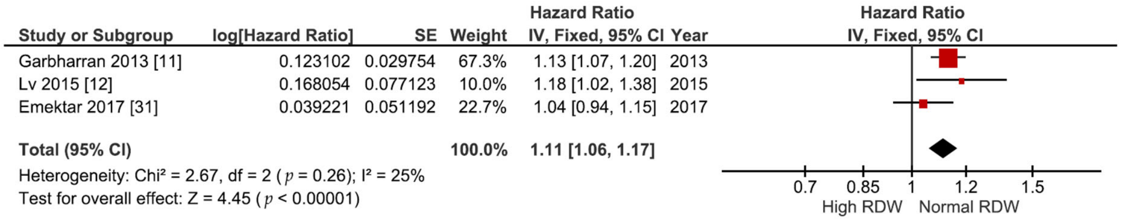

Figure S1. Forest plot of the association between RDW and >1-year mortality risk after hip fractures

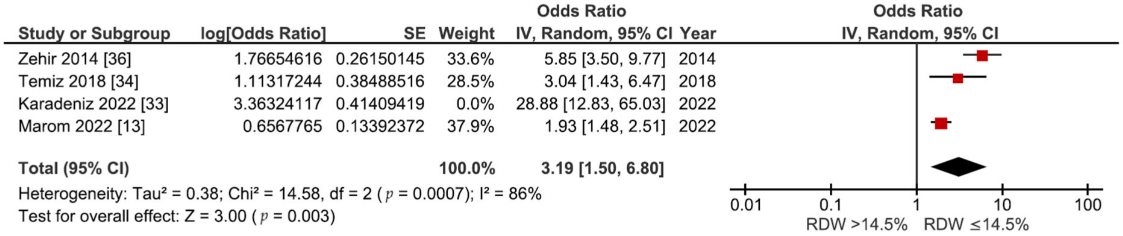

Figure S2. Forest plot of the association between RDW and 1-year mortality risk following hip fractures after sensitivity analysis

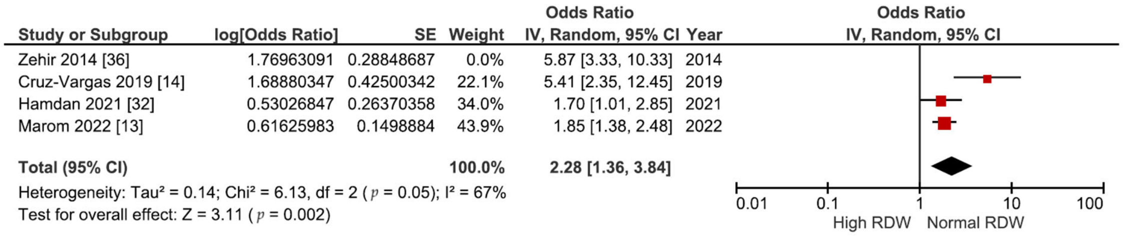

Figure S3. Forest plot of the association between RDW and 6-month mortality risk following hip fractures after sensitivity analysis
